# A series of cases of transthyretin amyloid cardiomyopathy with negative bone scintigraphy but a confirmed positive endomyocardial biopsy

**DOI:** 10.1186/s13023-024-03401-9

**Published:** 2024-10-15

**Authors:** Antoine Fraix, Emmanuel Itti, Amira Zaroui, Mounira Kharoubi, Elsa Poullot, Lionel Lerman, Soulef Guendouz, Olivier Huttin, Thibaud Damy, Arnault Galat

**Affiliations:** 1 Cardiology Department, Institut Lorrain du Cœur et des Vaisseaux, Nancy University Medical Center, 54000 Nancy, France; 2 AP-HP (Assistance Publique-Hôpitaux de Paris), Nuclear Medicine Department, Henri Mondor University Medical Center, 51 Avenue du Maréchal de Lattre de Tassigny, 94010 Créteil, France; 3 AP-HP (Assistance Publique-Hôpitaux de Paris), Cardiology Department, Henri Mondor University Medical Center, 51 Avenue du Maréchal de Lattre de Tassigny, 94010 Créteil, France; 4 AP-HP (Assistance Publique-Hôpitaux de Paris), French National Reference Center for Cardiac Amyloidosis, Cardiogen Network, Henri Mondor University Medical Center, 51 Avenue du Maréchal de Lattre de Tassigny, 94010 Créteil, France; 5 AP-HP (Assistance Publique-Hôpitaux de Paris), GRC Amyloid Research Institute, Henri Mondor University Medical Center, 51 Avenue du Maréchal de Lattre de Tassigny, 94010 Créteil, France; 6 AP-HP (Assistance Publique-Hôpitaux de Paris), DHU A-TVB, Henri Mondor University Medical Center, 51 Avenue du Maréchal de Lattre de Tassigny, 94010 Créteil, France; 7 IMRB, Inserm, University Paris Est Créteil, 94000 Creteil, France; 8 Pathology Department, AP-HP (Assistance Publique-Hôpitaux de Paris), Henri Mondor University Medical Center, 51 Avenue du Maréchal de Lattre de Tassigny, 94010 Créteil, France

**Keywords:** Transthyretin amyloid cardiomyopathy, Negative bone scintigraphy, Biopsy, Mutation, Case series

## Abstract

**Background:**

Bone scintigraphy (BS) is established as an accurate, non-invasive method for the diagnosis of transthyretin amyloid cardiomyopathy (ATTR-CM). In a real-life setting, however, some patients with no cardiac uptake on BS turn out to have cardiac-biopsy-confirmed ATTR-CM. We retrospectively included all patients diagnosed at the French Referral Center for ATTR-CM and who had data for BS and a cardiac biopsy.

**Results:**

Of 271 patients with positive cardiac biopsy, 14 (5%) had no cardiac uptake on ^99m^Tc-hydroxymethylene diphosphonate BS. Cardiac uptake was found in four of the seven patients who had a second BS assessment with ^99m^Tc-3,3-diphosphono-1,2-propanodicarboxylic acid (DPD). A retrospective review of the BS data found low cardiac uptake in four patients (two with HMDP and two with both radiotracers). Ultimately, six of the 14 patients with a biopsy-confirmed diagnosis of ATTR-CM did not show any cardiac radiotracer uptake.

**Conclusions:**

An endomyocardial biopsy may be necessary for confirming the diagnosis of ATTR-CM in patients with clinical and imaging signs of cardiac amyloidosis but no cardiac radiotracer uptake in BS.

## Introduction

Transthyretin amyloid cardiomyopathy (ATTR-CM) is an emerging cause of heart failure. Recent advances in non-invasive diagnosis and greater awareness among cardiologists have increased the frequency with which ATTR-CM is diagnosed. As recommended in a consensus statement by the European Society of Cardiology, bone scintigraphy (BS) with three ^99m^technetium (^99m^Tc)-labeled radiotracers (^99m^Tc-hydroxymethylene diphosphonate (HMDP), ^99m^Tc-3,3-diphosphono-1,2-propanodicarboxylic acid (DPD) and ^99m^Tc-pyrophosphate (PYP)) is used to diagnose of cardiac amyloidosis. Once light chain amyloidosis has been ruled out, the myocardial uptake of these radiotracers has high diagnostic value for ATTR-CM [1, 2].

Even though most diagnoses of ATTR-CM are based on non-invasive techniques, biopsies of the accessory salivary glands and (less frequently) the endomyocardium are essential when the BS results are inconclusive or when gammopathy is present. Recent studies have suggested that the absence of cardiac radiotracer uptake (i.e. a Perugini score of 0) can rule out ATTR-CM with 100% accuracy [3]. However, following the publication of Rauf et al*.*’s results, we observed that some of our patients with clinically suspected ATTR-CM (subsequently confirmed by a cardiac biopsy) did not present cardiac uptake at BS.

Hence, the objective of the present real-life study was to describe the diagnostic work-up and characteristics of patients with negative BS results but a histologically confirmed diagnosis of ATTR-CM.

## Materials and methods

### Study population and diagnostic work-up

**Fig. 1:**
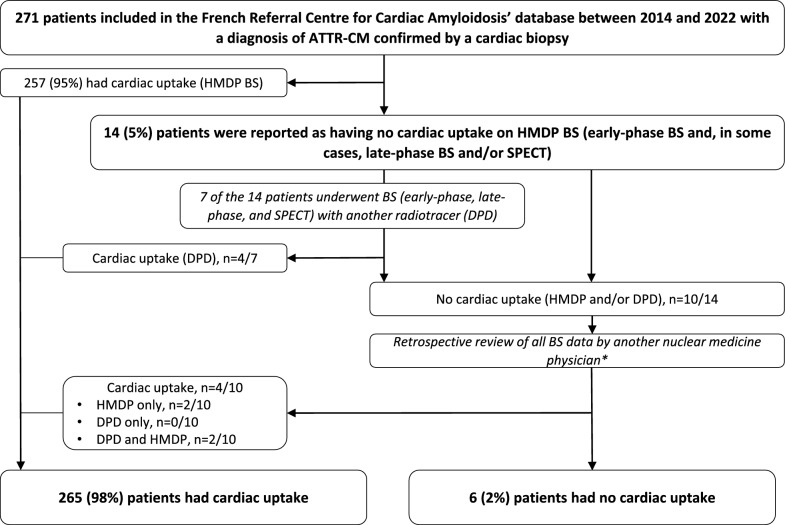
Study flow chart. *ATTR-CM* transthyretin amyloid cardiomyopathy; *BS* bone scintigraphy; *DPD* 99mTc-3,3-diphosphono-1,2-propanodicarboxylic acid; *HMDP* hydroxymethylene diphosphonate-99 Technetium; *SPECT* single photon emission computed tomography * All previously described cases of cardiac uptake were confirmed

We retrospectively analyzed data on patients consulting at the French Referral Center for Cardiac Amyloidosis (Henri Mondor University Medical Center, Créteil, France) between November 5th, 2014, and December 7th, 2022 (Fig. 1). All the patients had been referred for suspected cardiac amyloidosis (CA) and had undergone a physical examination, electrocardiography, transthoracic echocardiography (TTE), cardiac MRI (when not contraindicated), serum biomarker assays (troponin T, N-terminal prohormone of brain natriuretic peptide, and creatinine), genetic sequencing of the transthyretin gene *TTR*, a salivary gland biopsy, and ^99m^Tc-HMDP-BS as part of their standard diagnostic work-up. We included all patients with a confirmed diagnosis of ATTR-CM, no cardiac radiotracer uptake in the first available BS, and a biopsy-confirmed final diagnosis of ATTR-CM. When CA was strongly suspected, a cardiac biopsy was performed even when the BS and the salivary gland biopsy were negative. Occasionally, BS with a different radiotracer was performed at the same time. In a few cases, a cardiac biopsy was sampled during cardiac surgery.

### Bone scintigraphy

In our center, ^99m^Tc-HMDP-BS was initially performed with late-phase acquisition (3 h) and single photon emission computed tomography (SPECT). Due to the high number of patients referred to our center and the patients’ frailty and old age, we had developed and validated an early-phase (10 min) acquisition technique and showed that a heart-to-mediastinum (H/M) ratio greater than 1.21 was diagnostic for ATTR-CM once gammapathy had been ruled out [4]. For research purposes (e.g. screening for inclusion in a trial) or if the results were inconclusive, ^99m^Tc-DPD-BS was performed with a late-phase (3 h) acquisition and SPECT (the Perugini score can only be determined on late-phase images). The absence of cardiac uptake in the initial BS was used to select patients for the present study. To confirm the results, all available BS data were reviewed retrospectively by a second nuclear medicine physician with an expertise in the diagnosis of CA.

### Cardiac biopsy

Our assessment of the cardiac biopsy included a macroscopic analysis after staining with hematoxylin–eosin, Congo Red, and Sirius Red. Positive samples were screened for yellow-green dichroism. Next, immunohistochemical and immunofluorescence typing of amyloid TTR, kappa and lambda deposits was performed. In difficult-to-interpret cases, mass spectrometry proteomic testing was also performed [5, 6].

## Results

### Characteristics of the study population

In the cohort as a whole, 271 patients had a biopsy-confirmed diagnosis of ATTR-CM. Fourteen patients (5%) had a negative initial ^99m^Tc-HMDP-BS and were included in our study (Fig. 1 and Table 1). Among them, 7 patients underwent another BS with a different radiotracer (^99m^Tc-DPD) and the presence of a low cardiac uptake was described in 4 cases.

**Table 1:** Baseline characteristics of the 14 patients with an initial negative BS result

Patient	*TTR* variant	Male sex	Age, years	Neuropathy/dysautonomia	CTS	SR	Troponin T hs, ng/L	NT-proBNP, ng/L	IVST, mm on TTE	LVEF (%) on TTE	LGE on CMR	EC biopsy
#1	*V30M* +/−	−	43	+/+	−	+	18	112	8	65	−	NA
#2	*V30M* +/−	−	44	+/+	−	+	21	419	8	53	−	NA
#3	0	+	78	+/+	−	−	27	2469	14	60	+	+ (SG)
#4	0	+	82	−/−	−	−	117	7223	19	43	+	+ (SG)
#5	*V122I* +/−	+	65	−/+	+	+	67	6966	15	45	+	NA
#6	*P133T* +/−	+	58	+/−	−	+	33	562	11	60	+	+ (SG)
#7	*V122I*+/+	−	72	+/−	−	−	69	871	14	60	NA	+ (SG)
#8	*V122I* +/−	−	81	+/−	+	−	20	416	16	66	NA	NA
#9	0	+	68	+/−	+	+	24	141	14	63	NA	− (SG)
#10	0	+	84	−/−	+	−	73	5575	23	50	+	− (SG)
#11	0	+	85	−/−	−	−	75	2460	14	54	+	− (SG)
#12	0	+	75	−/−	−	+	136	9019	15	20	NA	− (SG)
#13	NA	+	90	−/−	−	+	78	11.886	20	62	NA	NA
#14	0	−	85	−/−	+	−	53	828	15	64	+	+ (SG)

*ATTR-CM* transthyretin amyloid cardiomyopathy; *CTS* carpal tunnel syndrome; *CMR* cardiac magnetic resonance; *EC* extracardiac; *hs* high sensitivity; *IVST* interventricular septal thickness; *LGE* late gadolinium enhancement; *LVEF* left ventricle ejection fraction; *NA* not available; *SG* salivary gland; *SR* sinus rhythm; *TTR* transthyretin

**Fig. 2:**
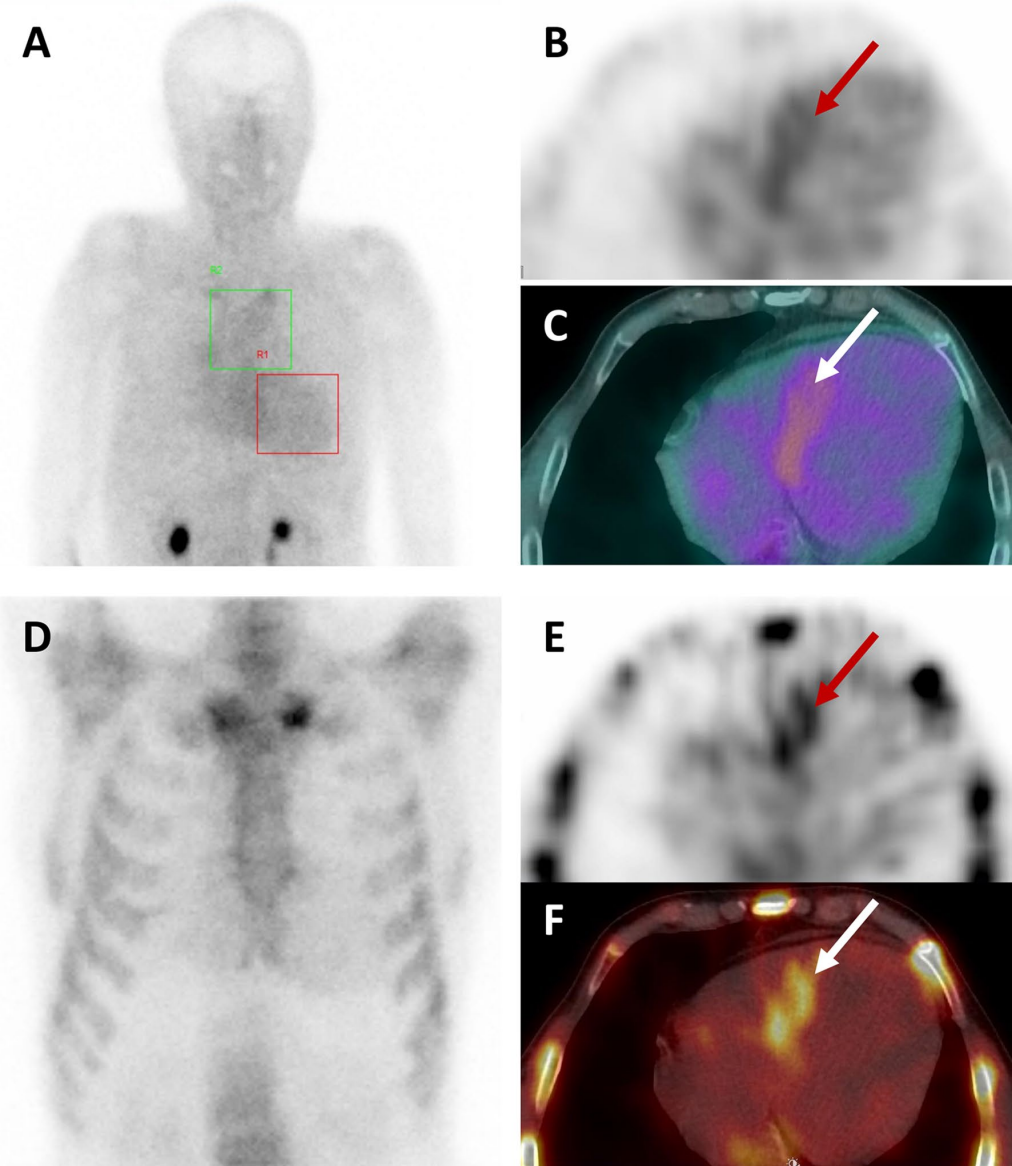
An example of false negative bone scintigraphy (patient #12). **A** Early ^99m^TC-HMDP-BS planar imaging with no cardiac uptake and an H/M ratio of 1.02. **B, C** Early ^99m^TC-HMDP-BS SPECT imaging (**B**) and coupled CT (**C**), showing basal septal uptake (red and white arrows). **D** Late ^99m^TC-HMDP-BS planar imaging with no cardiac uptake **E, F** Late ^99m^TC-HMDP-BS SPECT imaging (**E**) coupled with CT (**F**), showing the same basal septal uptake (red and white arrows)

**Fig. 3:**
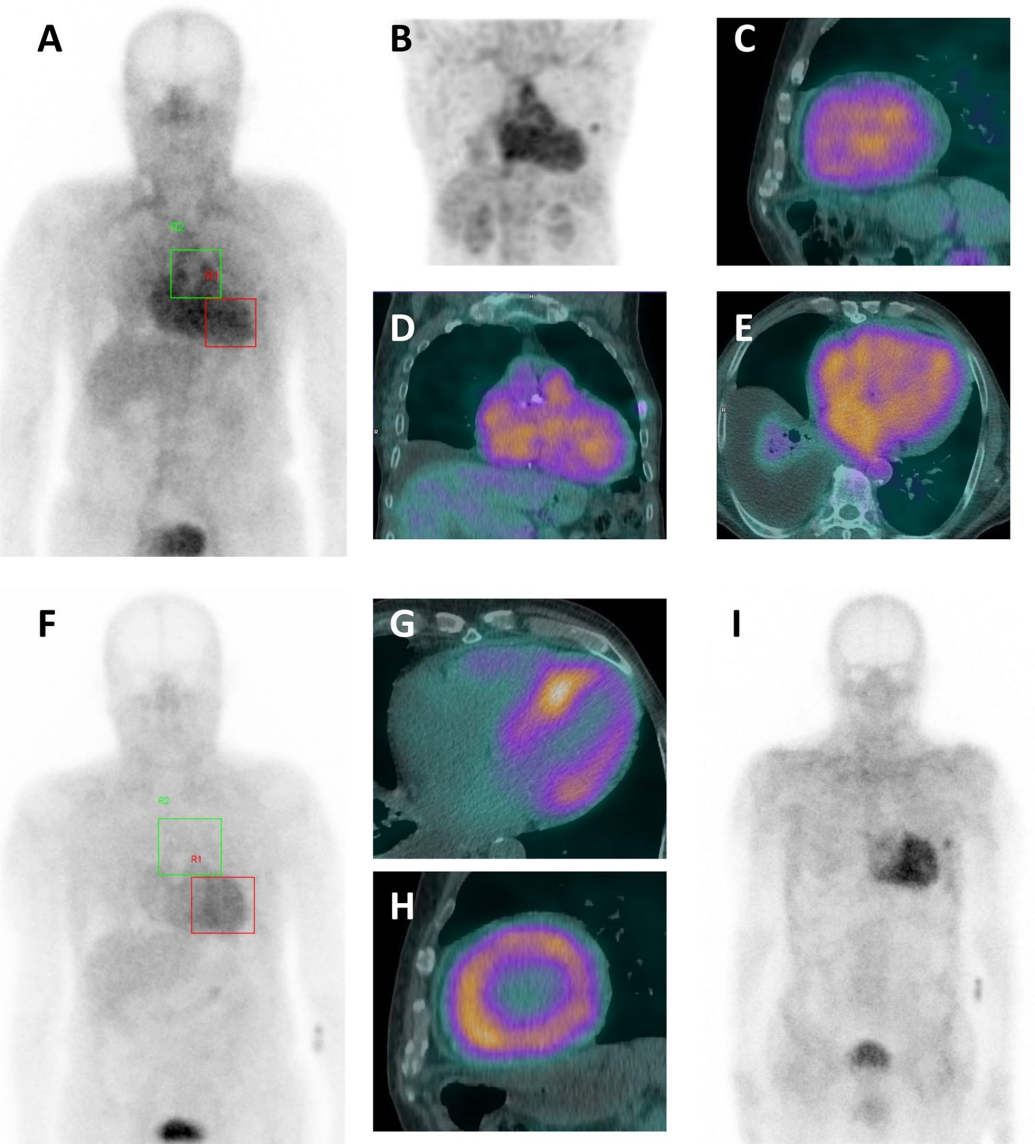
An example of true negative HMDP bone scintigraphy but positive DPD bone scintigraphy (patient #10). **A, B** Early ^99m^TC-HMDP-BS planar imaging (**A**) and a maximum intensity projection sequence (**B**) presenting diffuse uptake with an H/M ratio of 1.01. **C–E** Early ^99m^TC-HMDP-BS SPECT imaging and coupled CT, showing diffuse uptake by the blood pool but no cardiac uptake. **F** Early ^99m^TC-DPD-BS planar imaging with cardiac uptake and an H/M ratio of 1.56. **G, H** Early ^99m^TC-DPD-BS SPECT imaging and coupled CT, showing diffuse myocardial uptake. **I** Late ^99m^TC-DPD-BS planar imaging with cardiac uptake (Perugini score: 3)

All patients’ BS results were subsequently reviewed by a different nuclear medicine physician. Of the 10 patients initially considered to be negative, 4 had a very mild basal septal uptake on HMDP (mainly visible with SPECT; Fig. 2) among them 2 patients were also positive on ^99m^Tc-DPD-BS after careful review. Surprisingly, one patient was truly negative in an early ^99m^HMDP scan and clearly positive in a ^99m^DPD scan (Fig. 3).

**Fig. 4:**
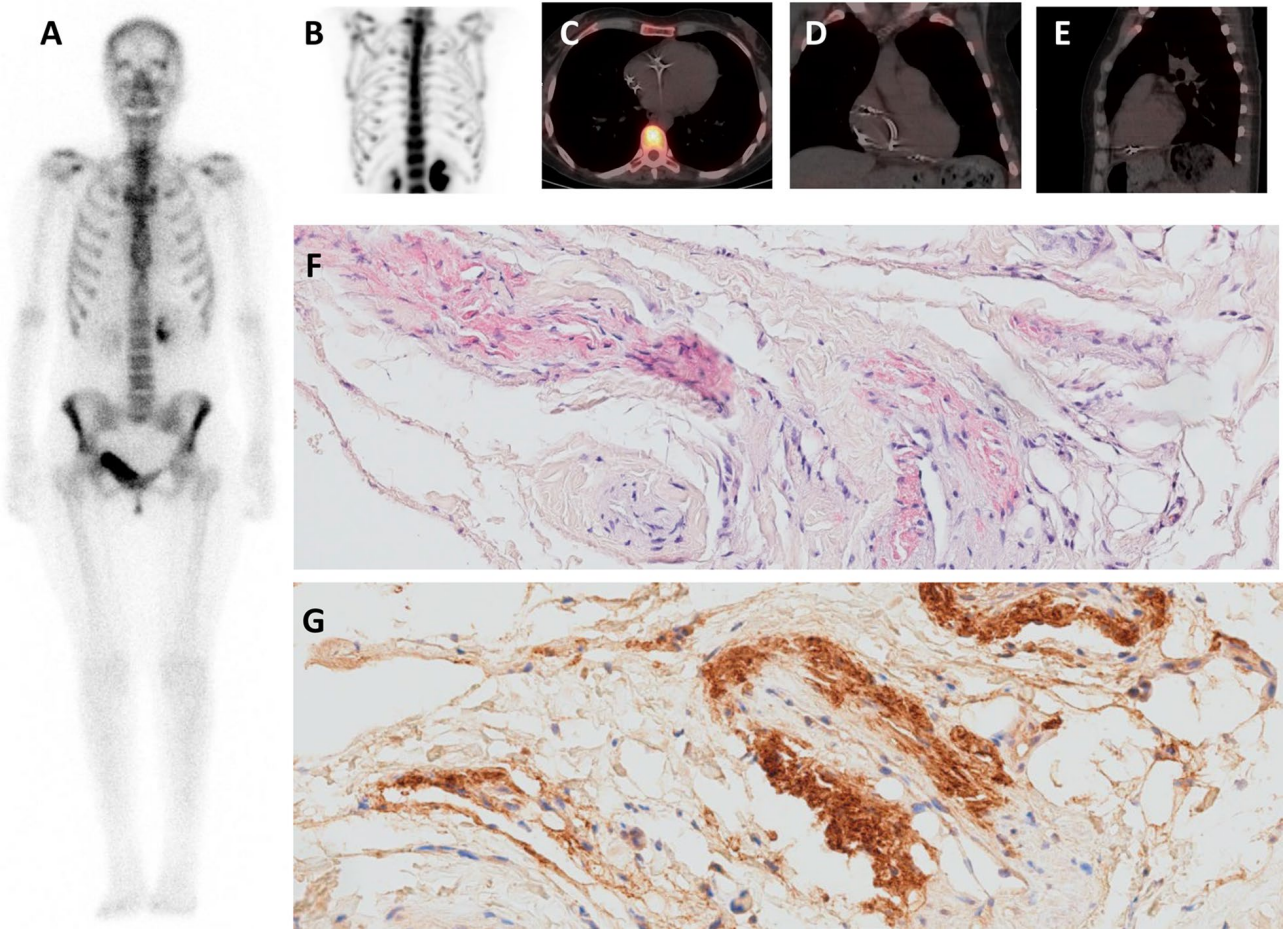
An example of true negative DPD bone scintigraphy (patient #1). **A, B** Late 99mTC-HMDP-BS planar imaging (**A**) and a maximum intensity projection sequence (**B**), with no cardiac uptake (Perugini 0). **C–E** Late 99mTC-HMDP-BS SPECT imaging and coupled CT, with no cardiac uptake. **F** Cardiac biopsy with positive Congo Red staining, indicating the presence of amyloid deposits **G** Cardiac biopsy with immunohistochemical and immunofluorescence typing of amyloid TTR deposits

Ultimately, six of the 14 patients were considered to have a true negative BS after a review of the initial result and then BS with a second radiotracer (Fig. 4). This corresponded to a BS false-negative rate of 2% among patients with biopsy-confirmed ATTR-CM.

### ***Description of the patients with a “false negative” BS for ATTR-CM diagnosis (***Tables 1 and 2***)***

**Table 2:** Characteristics of the six patients with a confirmed diagnosis of ATTR-CM and truly negative BS

Patient	*TTR* variant	Age (years)	HMDP-BS Early/late phase	DPD-BS Early/late phase	Type of cardiac biopsy	CRSR	Type of amyloid deposit	Immunostaining	Mass spectrometry—proteomics
#1	*V30M* +/−	43	−/−	NA	P	+	I–V	TTR:++ Kappa: − Lambda: −	NA
#2	*V30M* +/−	44	−/NA	NA	A	+	N–I	TTR:+ Kappa: − Lambda: −	NA
#3	0	78	−/NA	NA	M	+	N–I	TTR:+++ Kappa:++ Lambda: −	TTR
#4	0	82	−/NA	NA	M	+	N–I	TTR:+++ Kappa: − Lambda: −	NA
#5	*V122I* +/−	65	−/NA	NA	M	+	N–I	TTR:+++ Kappa: − Lambda: −	NA
#6	*P133T* +/−	58	−/NA	NA/−	M	+	V	NA	TTR

*A* atrial; *BS* bone scintigraphy; *CRSR* Congo Red-Sirius Red staining; *DPD* 3,3-diphosphono-1,2-propanodicarboxylic acid; *HMDP* hydroxymethylene diphosphonate; *I* interstitial; *M* myocardium; *N* nodular; *NA* not available; *P* pericardial; *TTR* transthyretin; *V* vascular

#### Patient #1

Patient 1 (a women born in 1971 and aged 43 at the time of writing) had been diagnosed with ATTRV30M neuropathy in 1994. She received a liver transplant in 2006. The cardiac assessments were inconclusive until 2014, when high-grade atrioventricular block prompted the placement of a pacemaker. This procedure was complicated by pneumothorax and pericardial effusion requiring surgery. A pericardial biopsy stained positive with Congo Red-Sirius Red (CRSR +), showed vascular deposits and was positive for TTR but negative for lambda and kappa chains. Early- and late-phase ^99m^Tc-HMDP-BS was negative (H/M ratio: 1; Perugini score: 0) (Fig. 4).

#### Patient #2

The patient (a man born in 1973 and aged 44 at the time of writing) was being followed up for symptomatic ATTRV30M neuropathy (diagnosed in 2001). The observation of a long His-to-ventricle interval with infraHisian atrioventricular block prompted the implantation of a pacemaker in 2009. In 2017, a comprehensive cardiac assessment included ^99m^Tc-HMDP-BS (negative; H/M ratio: 0.89), cardiac CT (negative, with no late enhancement), and TTE (inconclusive, mainly due to the absence of left ventricle (LV) hypertrophy). In 2019, the patient presented with an episode of right heart failure, due to a massive tricuspid regurgitation (associated with the pacemaker probes). The original pacemaker was replaced with an epicardial pacemaker, and the tricuspid valve was repaired. A surgical left atrial biopsy was CRSR + , had nodular deposits and was positive for TTR but negative for lambda and kappa chains.

#### Patient #3

This patient (a man born in 1938 and aged 78 at the time of writing) was being monitored for arrhythmia-induced cardiomyopathy with atrial fibrillation. Sinus node dysfunction prompted the implantation of a pacemaker in 2015. Furthermore, dysautonomia and severe orthostatic hypotension were diagnosed in 2015. In 2016, cardiac MRI with lateral intramyocardial late gadolinium enhancement was unspecific for ATTR-CM. In the same year, a duodenal biopsy collected during an endoscopic exploration was found to be positive for ATTR. There was no cardiac uptake of ^99m^Tc-HMDP in BS (negative; H/M ratio: 1.12) but the TTE findings were coherent with ATTR-CM (including LV hypertrophy and apical sparing). Gammopathy was ruled out, and genetic testing did not reveal any pathogenic variants. An initial accessory salivary gland biopsy showed a slight vascular amyloid deposit (CRSR +) that was positive for TTR and negative for lambda and kappa chains. An endomyocardial biopsy was CRSR + , had nodular deposits and was positive for TTR and kappa chains but negative for lambda chains. Additional proteomic testing confirmed the presence of ATTR deposits.

#### Patient #4

This patient (a man born in 1940 and aged 82 at the time of writing) was primarily being monitored for arrhythmia-induced cardiomyopathy with embolic atrial fibrillation. In 2022, he was admitted to another hospital for heart failure and tachy-brady syndrome; this led to the implantation of a pacemaker. The results of TTE and cardiac MRI at this time prompted us to suspect ATTR-CM. Further TTE in our hospital confirmed this suspicion, with concentric LV hypertrophy and apical sparing. ^99m^Tc-HMDP-BS was negative (H/M ratio: 1.0). Gammopathy was ruled out by laboratory tests, and genetic testing did not reveal any pathogenic variants. An initial accessory salivary gland biopsy showed slight interstitial amyloid deposits (CRSR +) but could not be typed. An additional endomyocardial biopsy was CRSR + , had nodular deposits and was positive for TTR and negative for lambda and kappa chains.

#### Patient #5

The patient (a man born in 1957 and aged 65 at the time of writing) presented at another hospital with an initial episode of heart failure in 2022. TTE showed bilateral ventricular hypertrophy, a somewhat low left ventricular ejection fraction (LVEF, 47%), and typical apical sparing. In our hospital, a further TTE assessment confirmed the suspected ATTR-CM, and cardiac MRI confirmed the slight deterioration of the LV ejection fraction (45%), an elevated extracellular volume (62%), and diffuse subendocardial late gadolinium enhancement suggestive of amyloid infiltration. Laboratory tests showed monoclonal IgG kappa gammopathy, in the absence of Bence Jones proteinuria. There was no cardiac uptake on ^99m^Tc-HMDP-BS (H/M ratio: 1.02). An endomyocardial biopsy showed nodular and interstitial deposits of amyloidosis (CRSR +) and was positive for TTR but negative for lambda or kappa chains. Genetic testing confirmed the presence of the ATTRV122I pathogenic variant.

#### Patient #6

The patient (a man born in 1953 and aged 58 at the time of writing) was admitted to hospital for heart failure in 2011. TTE revealed dilated cardiomyopathy, a low LVEF (33%), new onset atrial fibrillation, and no coronary artery disease. The disease progressed rapidly; the LVEF fell to 15%, prompting the implantation of a biventricular assist device. A surgical myocardial biopsy showed vascular nontyped amyloid deposits. In 2012, the patient received a heart transplantation. An analysis of explanted heart tissue revealed diffuse amyloid deposits. Genetic testing showed the presence of ATTRP133T, variant of uncertain significance. Extracardiac involvement was confirmed histologically on salivary gland and liver biopsies. A proteomic analysis confirmed the ATTR-CM and the presence of the ATTRP133T variant in the salivary gland. In 2019, the TTE findings were consistent with early transplant recurrence of ATTR-CM. Although the transplanted heart did not take up ^99m^Tc-HMDP (H/M ratio: 1.05) or ^99m^Tc-DPD (Perugini 0), an endomyocardial biopsy confirmed the myocardial and pericardial recurrence of vascular ATTR deposits. This recurrence of ATTR-CM after heart transplantation was treated with tafamidis.

## Discussion

The case descriptions given above emphasize how difficult it is to confirm the presence of ATTR-CM in some patients. Although ATTR-CM is diagnosed by non-invasive imaging (i.e. positive BS in the absence of gammopathy) in most cases, an endomyocardial biopsy is sometimes mandatory—particularly when CA is very likely and other tests (biopsies of extracardiac tissue, BS, etc.) are inconclusive. Here, we included 14 patients with initially negative ^99m^Tc-HMDP-SPECT-BS and a positive cardiac biopsy. After a second BS assessment (with ^99m^Tc-DPD) and a retrospective review, we confirmed the absence of cardiac radiotracer uptake in six of the 14 patients.

### How can we improve the sensitivity of BS in the diagnosis of ATTR-CM?

When the BS is negative but the results of other tests (TTE, cardiac MRI, etc.) are strongly suggestive of CA, a number of simple, non-invasive options are available.

Firstly, the BS results should be reviewed with an expert nuclear medicine physician. Although the interpretation of BS data might appear to be easy at first sight, our results show that even patients consulting in a referral center can be misclassified. Here, four patients were reclassified by the reviewer as having a positive BS result on HMDP scan. This proportion is nevertheless very small, given the number of BSs performed during the study period (2965 BSs between November 5th, 2014, and December 7th, 2022). To improve the test’s accuracy, we sought to highlight the diagnostic importance of concomitant SPECT imaging; as shown in Fig. 2, cardiac uptake was sometimes located only in basal segments (especially in patients with early-stage disease) and could be hidden by the sternum [7].

Secondly, additional BS can be performed with a different radiotracer. In our center, we use early-phase ^99m^Tc-HMDP-BS (planar + SPECT) in routine practice but we switch to early- and late-phase ^99m^Tc-DPD BS (planar + SPECT) for inconclusive findings or for research purposes. As mentioned above, the Perugini score can only be determined for late-phase imaging. An additional BS examination confirmed the diagnosis of ATTR-CM in six of our seven suspected cases. To the best of our knowledge, the various radiotracers have not been compared in large cohort studies. Only a few patients have different results for ^99m^Tc-DPD, ^99m^Tc-HMDP vs. ^99m^PYP radiotracers [8]. In difficult-to-interpret cases, a comprehensive BS assessment (early- and late-phase protocols, with planar and SPECT data) with two different radiotracers must be considered and might prompt the physician to prescribe additional tests (such as an endomyocardial biopsy). It is important to discuss the modalities of BS and additional diagnostic options with an expert nuclear medicine physician.

### How can we explain a true negative BS result?

The lack of cardiac radiotracer uptake in BS cannot completely rule out ATTR-CM. One of the best-known causes of a negative BS result is the presence of pathogenic *TTR* variants – primarily ATTRV30M and ATTRP64L [9–11]. In our cohort of six patients with true negative BS, four carried a pathogenic *TTR* variant. Patients #1 and #2 carried the conventional early-onset ATTRV30M mutation. One can hypothesize that the low cardiac radiotracer uptake in cases of ATTRV30M cardiomyopathy is due to the presence of type B fibrils [12]. Unlike the more common type A fibrils, type B fibrils appear to have a lower affinity for conventional radiotracers.

Patient #6 carries a rare ATTRP133T mutation, which leads to systemic vascular amyloidosis with heart, liver, and salivary gland involvement. The type and localization of the amyloid deposits might also influence the scintigraphy results because vascular deposits may be associated with low or no cardiac uptake, relative to interstitial deposits. In our population, two patients (#1 and #6) presented with vascular deposits and two (#1 and #2) had biopsies collected from unusual locations (the pericardium or the atrium). These cases challenge the definition of CA. Atypical types or sites of deposit might lead to an erroneous non-invasive diagnosis.

However, the three other patients (#3 to #5) were not expected to have a negative BS result. Indeed, patients #3 and #4 did not have a genetic variant, and patient #5 had a ATTRV122I variant not typically associated with a negative BS result The deposits were always interstitial (whether nodular or not). Martini et al*.* also described a middle-aged patient with a TTR-positive endomyocardial biopsy and negative ^99m^Tc-DPD-BS [13]. Genetic tests were negative, and the deposits were extracellular. As long as the determinants of the cardiac uptake of bone radiotracers in ATTR-CM have not been defined, questions will remain.

### A practical approach for confirming the diagnosis of ATTR-CM when the BS is negative

When the clinical and echocardiographic data are strongly suggestive of CA but BS is negative, the first step is to confirm the negative results by reviewing the BS data with an expert nuclear medicine physician. Secondly, it is important to ensure that a comprehensive BS protocol (with SPECT and late-phase imaging) was applied. Lastly, one can consider a second BS assessment with SPECT, late-phase imaging, and perhaps a different radiotracer. In any case, it is important to bear in mind that certain variants (such as ATTRV30M and P64L) are associated with negative BS results.

If the BS is still negative, only a biopsy can confirm the presence or absence of ATTR-CM. In such a case, an extracardiac biopsy (such as a salivary gland biopsy) is generally performed first and can be followed by an endomyocardial biopsy of the right ventricle (and, in some cases, the left ventricle additionally) if the diagnosis is still unclear. An endomyocardial biopsy usually provides a definitive diagnosis and, as shown by our present results, must not be overlooked. It is also important to remember that a mass spectrometry proteomic analysis may be applied if amyloid deposits cannot be typed with immunohistochemical and immunofluorescence techniques.

Our study had a number of limitations. Firstly, it was a retrospective study. Secondly, the study cohort was small. Nevertheless, this was the largest yet reported cohort of patients with ATTR-CM and a negative BS result. Thirdly, not all the patients underwent an additional BS with a comprehensive protocol (i.e. SPECT and late-phase imaging) or with another radiotracer. Lastly, BS and the endomyocardial biopsy were not performed at the same point in time. However, ATTR-CM is a slowly progressing disease, and so the time interval between BS and biopsy is unlikely to be a major source of bias.

## Conclusions

Our results emphasize how difficult it is to diagnose ATTR-CM and highlight the limitations of non-invasive techniques. Negative BS—even when the Perugini score is 0—should not formally rule out a diagnosis of clinically suspected ATTR-CM. In such cases, it is mandatory to review or repeat the BS and to perform additional tests (such as a cardiac biopsy) so that a patient with ATTR-CM can be treated specifically.

## Data Availability

The data underlying this article cannot be shared publicly due to the privacy of the study participants. The data will be shared on reasonable request to the corresponding author.

## Abbreviations

ATTR: Transthyretin amyloid protein
ATTR-CM: Transthyretin amyloid cardiomyopathy
BS: Bone scintigraphy
CA: Cardiac amyloidosis
CRSR: Congo Red-Sirius Red
DPD: 3,3-Diphosphono-1,2-propanodicarboxylic acid
H/M: Heart-to-mediastinum ratio
HMDP: Hydroxymethylene diphosphonate
LV: Left ventricle
PYP: Pyrophosphate
SPECT: Single photon emission computed tomography
TTE: Transthoracic echocardiography
99mTc: Technetium-99m
